# Epigenetic reprogramming promotes the antiviral action of IFNα in HBV-infected cells

**DOI:** 10.1038/s41420-021-00515-y

**Published:** 2021-06-02

**Authors:** Luc Gailhouste, Masayuki Sudoh, Xian-Yang Qin, Koichi Watashi, Takaji Wakita, Takahiro Ochiya, Tomokazu Matsuura, Soichi Kojima, Yutaka Furutani

**Affiliations:** 1 Liver Cancer Prevention Research Unit, RIKEN Cluster for Pioneering Research, Wako, Japan; 2 Department of Translational Research, Joint Research Center for Human Retrovirus Infection, Kagoshima University, Kagoshima, Japan; 3 Research Center for Drug and Vaccine Development, National Institute of Infectious Diseases, Tokyo, 162-8640 Japan; 4 Department of Virology II, National Institute of Infectious Diseases, Tokyo, Japan; 5 Department of Molecular and Cellular Medicine, Tokyo Medical University, Tokyo, Japan; 6 Department of Laboratory Medicine, The Jikei University School of Medicine, Tokyo, Japan

**Keywords:** DNA methylation, Viral infection

## Abstract

Chronic hepatitis B virus (HBV) infections remain a health burden affecting ~250 million people worldwide. Thus far, available interferon-alpha (IFNα)-based therapies have shown unsatisfactory cure rates, and alternative therapeutic molecules are still required. However, their development has been hampered because accessible cell models supporting relevant HBV replication and appropriate antiviral activity are lacking. Strategies that reverse epigenetic alterations offer a unique opportunity for cell reprogramming, which is valuable for restoring altered cellular functions in human cell lines. This work aimed to investigate the feasibility of converting HepG2 cells that stably overexpress the HBV entry receptor (sodium/taurocholate cotransporting polypeptide, NTCP) toward IFNα-responsive cells using epigenetic reprogramming. Herein, we showed that an epigenetic regimen with non-cytotoxic doses of the demethylating compound 5-azacytidine restored the anti-HBV action of IFNα in epigenetically reprogrammed HepG2-NTCP-C4 cells, named REP-HepG2-NTCP cells. Thus, a significant inhibition in HBV DNA levels was measured in REP-HepG2-NTCP cells after IFNα treatment. This inhibitory effect was associated with the enhancement of IFNα-mediated induction of critical interferon-stimulated genes (ISGs), which was limited in non-reprogrammed cells. In particular, our data indicated that re-expression of 2’-5’-oligoadenylate synthetase 1 (*OAS1*) and interferon regulatory factor 9 (*IRF9*) was the result of an epigenetically driven unmasking of these genes in reprogrammed cells. At last, we evaluated the therapeutic potential of the IFN analog CDM-3008 in REP-HepG2-NTCP cells and demonstrated the efficiency of this chemical compound in triggering ISG induction and HBV inhibition. In summary, this study shows that epigenetic reprogramming promotes the IFNα response in HBV-infected cells and is potentially attractive for cell-based experimental screening of IFN-like compounds.

## Introduction

Hepatitis B virus (HBV) infections represent a major public health issue. Despite effective vaccination, ~250 million individuals are chronically infected with the virus worldwide^1^. Without treatment, chronic HBV infections gradually progress to liver fibrosis and cirrhosis^2^. Liver cirrhosis is associated with an increased risk of hepatocellular carcinoma (HCC), one of the leading causes of cancer-related death^3^. Currently, HBV therapeutic strategies include two formulations of interferon-alpha (IFNα) and five nucleos(t)ide analogs^4^. Nucleos(t)ide analogs negatively modulate the viral polymerase and de novo synthesis of HBV DNA^5^, whereas IFNα interferes with viral replication by inducing multiple interferon-stimulated genes (ISGs), subsequently affecting various steps of the viral life cycle^6,7^. However, more effective therapies are still required because of the relatively low curative rates obtained. Notably, prolonged treatments with IFNα are associated with side effects, and cessation of nucleos(t)ide analog treatments is commonly accompanied by a virological relapse due to the inability of these molecules to eradicate HBV covalently closed circular DNA, which accumulates in hepatocyte nuclei and functions as a persistent template for virus production^8^.

Understanding the molecular mechanisms by which IFNα mediates its antiviral action is critical for the development of new potent IFN-like compounds. In addition to primary human hepatocytes (PHHs), various experimental cell culture systems have provided alternative and more accessible tools for the study of anti-HBV molecules^9–11^. Thus far, additional efforts have been required to obtain more-consistent models that achieve HBV infection/replication rates and IFN-induced anti-HBV responses similar to those observed in PHHs. HepG2 cells that stably overexpress the HBV entry receptor sodium/taurocholate cotransporting polypeptide (*NTCP*), also known as solute carrier family 10 member 1 (*SLC10A1*), represent an affordable and well-characterized model widely used in HBV research laboratories^12–14^. However, low ISG induction levels and limited or inconsistent anti-HBV activities frequently preclude the use of HepG2-NTCP cells for reliable anti-HBV drug-screening applications^15^. We previously reported that hepatic cell lines treated with low concentrations of the DNA-demethylating reagent 5-azacytidine (5-AZA) recovered gene expression profiles and functional features similar to hepatocytes^16,17^. The present work was based on the hypothesis that this procedure, named epigenetic reprogramming, can restore a consistent antiviral response in HepG2-NTCP cells through the re-expression of epigenetically silenced ISGs. Here, our results showed restoration of the IFNα response in epigenetically reprogrammed HepG2-NTCP-C4 (REP-HepG2-NTCP) cells, whereas IFNα had no effect in non-reprogrammed HepG2-NTCP-C4 cells. This IFNα response was correlated with an augmentation of major ISGs known to be associated with the antiviral effect of IFNα in patients. Methylation analysis revealed the epigenetic unmasking of critical ISGs in REP-HepG2-NTCP cells, which were silenced by hypermethylation before reprogramming. Finally, we evaluated the potential of epigenetically reprogrammed cells for new drug screening by assessing the anti-HBV action of an IFN-like chemical compound.

## Results

### Epigenetic reprogramming restores the IFNα response in HepG2-NTCP-C4 cells

**Fig. 1:**
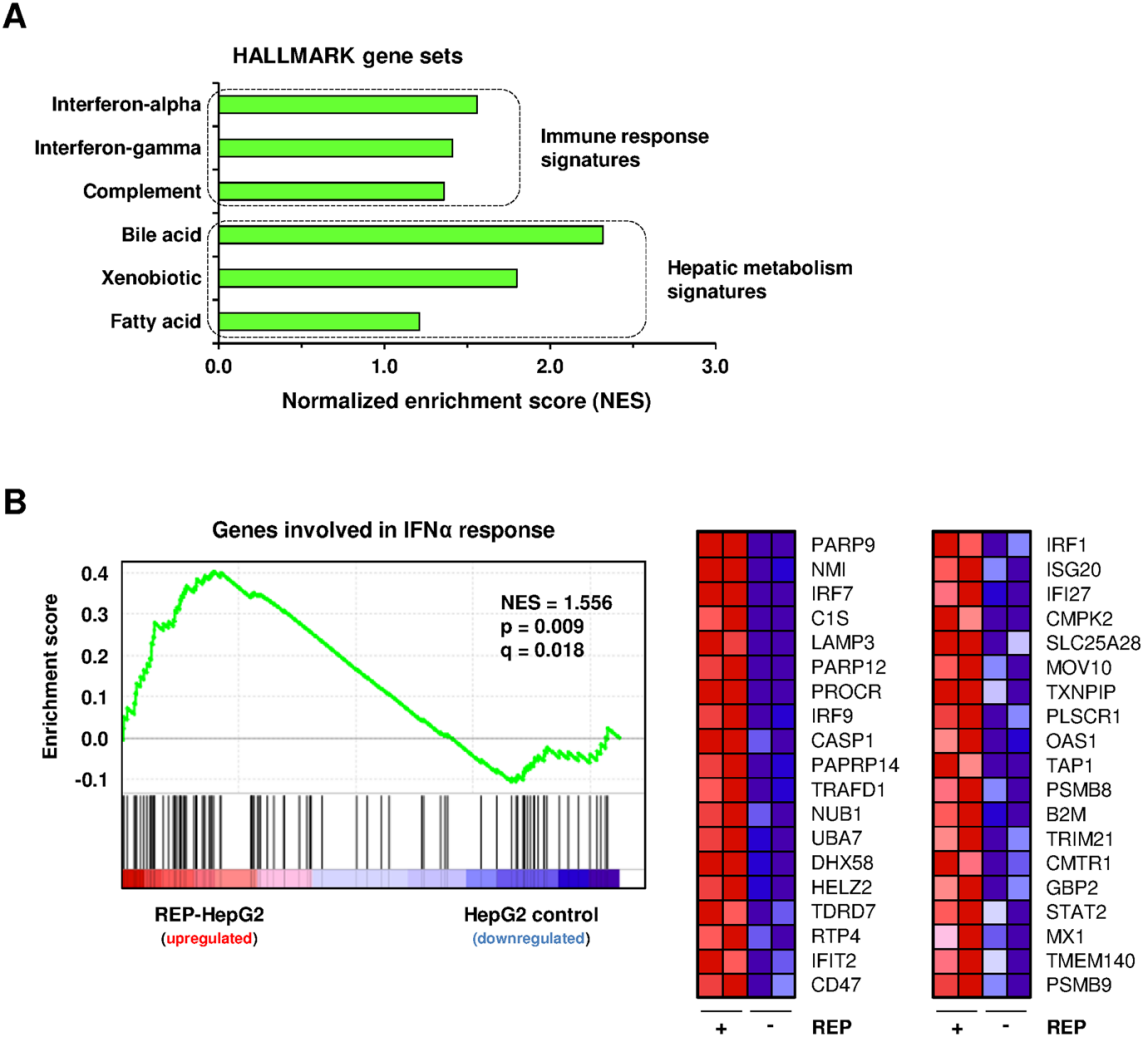
Gene set enrichment analysis (GSEA) after epigenetic reprogramming of HepG2 cells. **A** Relevant gene sets enriched in reprogrammed HepG2 (REP-HepG2) cells. Epigenetic reprogramming of HepG2 cells was achieved with a 2.5 µM 5-AZA regimen for 10 days. Untreated HepG2 cells were used as controls. The ranking of the depicted enriched gene sets was as follows: (#1) bile acid metabolism (molecular signature with the highest enrichment score in the HALLMARK gene datasets), (#3) xenobiotic metabolism, (#5) IFNα response, (#9) IFNγ response, and (#10) complement system. The enrichment of the hepatocyte metabolism signatures was consistent with the promotion of hepatic differentiation in the REP-HepG2 cells. The GSEA was performed using microarray data from the GSE160648 data set. **B** Enrichment plot and heat map for the genes involved in the IFNα response in the reprogrammed versus non-reprogrammed HepG2 cells (normalized enrichment score [NES] = 1.556; *p* = 0.009 and *q* = 0.018). See S. Table 1 for a complete list of the HALLMARK_interferon alpha genes. 1 mM 5-AZA = 244.2 ng/mL.

To explore the hypothesis that a demethylating treatment could promote cell antiviral activity, we first compared global gene expression profiles between 5-AZA-treated HepG2 cells and control HepG2 cells using microarray data from our previous work^16^. The results from a gene set enrichment analysis (GSEA) highlighted three signatures related to the immune response: (i) IFNα response, (ii) IFNγ response, (iii) and complement system (Fig. 1A). In particular, among the 90 genes that composed the IFNα response gene set, 38 genes appeared significantly enriched in HepG2 cells after epigenetic reprogramming (Fig. 1B), supporting a possible improvement of the antiviral response in these cells.

**Fig. 2:**
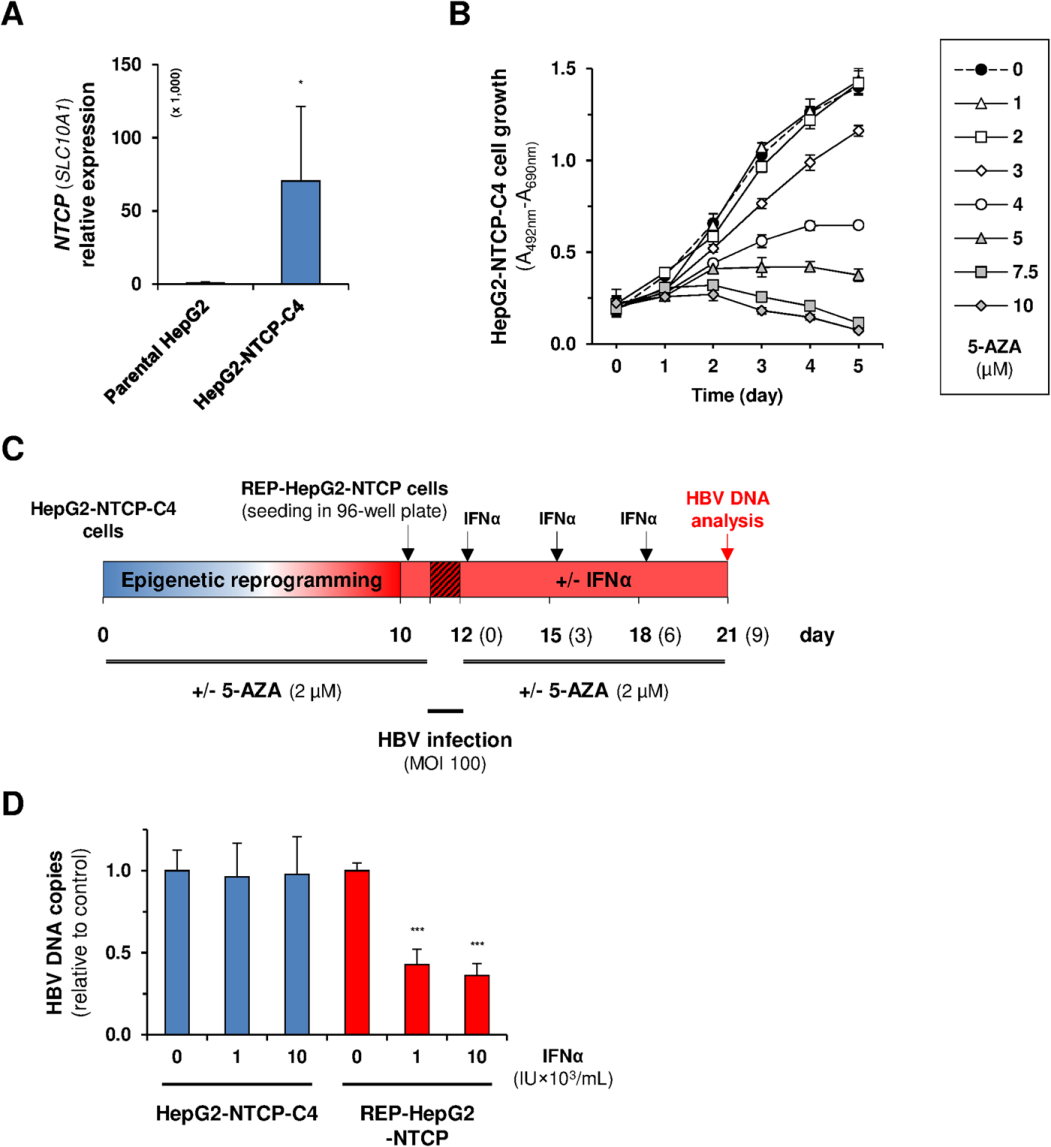
Epigenetic reprogramming of HepG2-NTCP-C4 cells and evaluation of IFNα antiviral activity after HBV infection. **A** Relative expression level of *NTCP* (*SLC10A1* gene) in HepG2 and HepG2-NTCP-C4 cells. *NTCP* was analyzed in samples from HepG2-NTCP-C4 cell cultures at different time-point (*n* = 6). Parental HepG2 cells were used as controls (*n* = 4). The histograms show the means ± SD. *p < 0.05 (*t* test). **B** Evaluation of 5-AZA cytotoxic effect on HepG2-NTCP-C4 cells. Twenty-four hours after seeding, the cells were treated with 5-AZA at the indicated concentrations for 5 days. HepG2-NTCP-C4 cell viability was determined at the indicated times using an XTT assay. The 5-AZA IC50 was 4.33 µM after 5 days of treatment. The data represent the means ± standard deviation (SD). **C** Epigenetic reprogramming experimental design. Cells were first treated with 2 µM 5-AZA for 10 days (reprogramming procedure). Twenty-four hours after seeding in 96-well plates, the reprogrammed HepG2-NTCP-C4 (REP-HepG2-NTCP) cells were infected with HBV at an MOI of 100 for 24 h. Next, REP-HepG2-NTCP cells were treated with 1,000 and 10,000 IU/mL IFNα every 3 days. Total DNA was collected for analysis after 9 days of treatment. The 5-AZA-supplemented medium was replaced daily during reprogramming and every 3 days after HBV infection. The numbers in brackets indicate the time after infection. No cytotoxic effect of IFNα was observed in the cells after treatment (S. Figure 1). **D** Measurement of HBV DNA levels in the HBV-infected cells after IFNα treatment. HBV DNA copies in the REP-HepG2-NTCP cells were determined by real-time quantitative PCR from 50 ng of total DNA. Histograms represent the means ± SD. Statistically significant differences in the HBV DNA levels related to untreated cells were achieved at ***p < 0.001 (*t* test).

Given the low susceptibly of the parental HepG2 cells for HBV infection, the HepG2-NTCP cell line was used in this study. In particular, we selected the HepG2-NTCP-C4 clone for epigenetic reprogramming because HepG2-NTCP-C4 cells exhibited high *NTCP* expression levels and high susceptibility to HBV infection, as previously described by Iwamoto and colleagues^13^. Before reprogramming, we confirmed the high expression levels of *NTCP* in HepG2-NTCP-C4 cells compared with the parental HepG2 cells (Fig. 2A). Our previous studies reported efficient epigenetic reprogramming in various cell lines using low doses of 5-AZA^16–18^. To determine the optimal concentration of the reagent for HepG2-NTCP-C4 epigenetic reprogramming, we analyzed cell viability after 5-AZA exposure. Our data showed a dose–response effect of the compound on cell growth, with marked cytotoxicity observed from 4 µM (Fig. 2B). In contrast, no significant cell toxicity or morphological alterations were observed at concentrations up to 2 µM after 5 days of treatment. Based on our previous work, a minimum of 7–10 days of treatment is usually essential to ensure sufficient demethylation and re-expression of epigenetically silenced genes in HCC cells using 5-AZA at non-cytotoxic doses^17^. Accordingly, we established a protocol for the epigenetic reprogramming of HepG2-NTCP-C4 cells based on a 2 µM 5-AZA regimen for 10 days with daily replacement of the medium.

To determine whether epigenetic reprogramming could stimulate IFNα antiviral activity, REP-HepG2-NTCP cells were infected with HBV using polyethylene glycol (PEG) 8000 at an MOI of 100 for 24 h after completing the reprogramming procedure, and HBV DNA levels were quantified following treatment with 1,000 and 10,000 IU/mL IFNα for 9 days (Fig. 2C). Importantly, we confirmed that IFNα and 5-AZA combination showed no cytotoxic effect in the cells after 9 days of treatment (S. Figure 1A). As expected, IFNα did not affect non-reprogrammed HepG2-NTCP-C4 cells, as the levels of HBV DNA remained unchanged after treatment (Fig. 2D). In contrast, the number of HBV DNA copies was reduced by 57.38% ± 0.09% and 63.97% ± 0.07% in REP-HepG2-NTCP cells in response to 1,000 and 10,000 IU/mL IFNα, respectively (*p* < 0.0001, *t* test), arguing for efficient restoration of the anti-HBV effect of IFNα in reprogrammed cells.

### Epigenetic reprogramming enhances the IFNα-mediated induction of ISGs

**Fig. 3:**
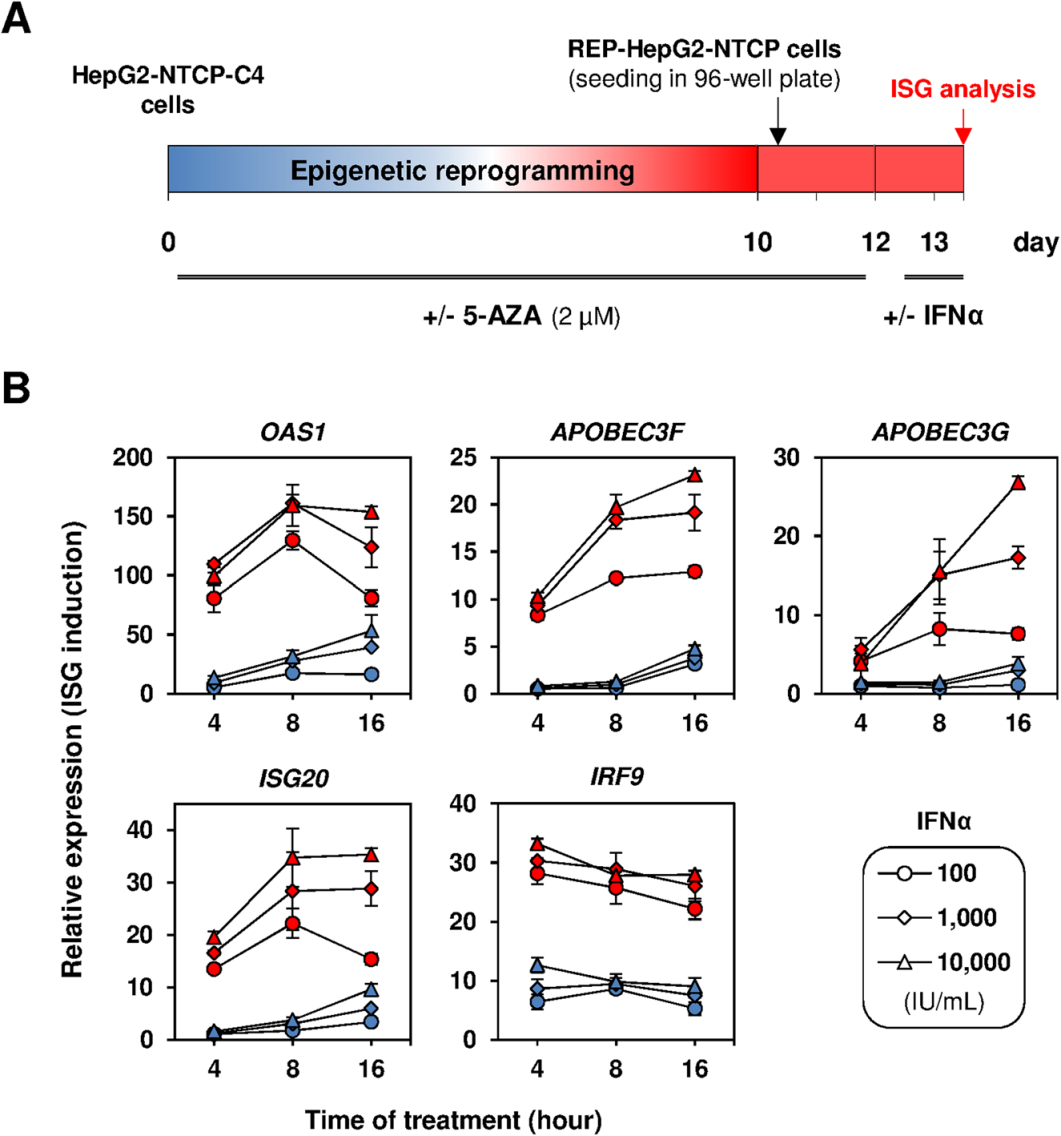
ISG expression analysis in IFNα-stimulated REP-HepG2-NTCP cells. **A** Experimental design. After epigenetic reprogramming using 2 µM 5-AZA for 10 days, REP-HepG2-NTCP cells were treated with different concentrations of IFNα for 4, 8, and 16 h. **B** Relative expression levels of 2’-5’-oligoadenylate synthetase 1 (*OAS1*), interferon-stimulated exonuclease gene 20 (*ISG20*), apolipoprotein B mRNA-editing enzyme catalytic subunits 3 F and 3 G (*APOBEC3F* and *APOBEC3G*), and interferon regulatory factor 9 (*IRF9*). Total RNA was extracted at the indicated times, and the relative gene expression levels were determined by real-time quantitative PCR. The data obtained from REP-HepG2-NTCP cells and naive HepG2-NTCP-C4 cells are depicted in red and blue, respectively, and show the means ± SD.

We assumed that HBV DNA inhibition after IFNα treatment in REP-HepG2-NTCP cells was a consequence of the re-expression of key ISGs in response to the demethylating treatment. To test this hypothesis, we evaluated the effect of IFNα on ISG expression in REP-HepG2-NTCP cells (Fig. 3A), with a special emphasis on critical ISGs previously reported to be associated with the IFNα-mediated antiviral response in patients^19^. Consistent with the GSEA data from the parental HepG2 cells (Fig. 1B), 2’-5’-oligoadenylate synthetase 1 (*OAS1*) and interferon-stimulated exonuclease gene 20 (*ISG20*) showed a significant augmentation in their expression, with level increases of 15.49 ± 2.20-fold and 12.32 ± 0.47-fold in REP-HepG2-NTCP cells compared with those in the non-reprogrammed cells after only 4 h of 100 IU/mL IFNα treatment (*p* = 0.0076 and *p* < 0.0001, respectively, *t* test) (Fig. 3B). Interestingly, the expression level of interferon regulatory factor 9 (*IRF9*), a transcription factor that controls the expression of numerous ISGs, was also increased by approximately threefold under these conditions. Finally, our data showed that the expression levels of apolipoprotein B mRNA-editing enzyme catalytic subunits 3 F and 3 G (*APOBEC3F* and *APOBEC3G*), two essential effectors of the anti-HBV response mediated by IFNα, were also markedly increased in the REP-HepG2-NTCP cells.

### IRF9 and OAS1 epigenetic silencing is abolished in REP-HepG2-NTCP cells

**Fig. 4:**
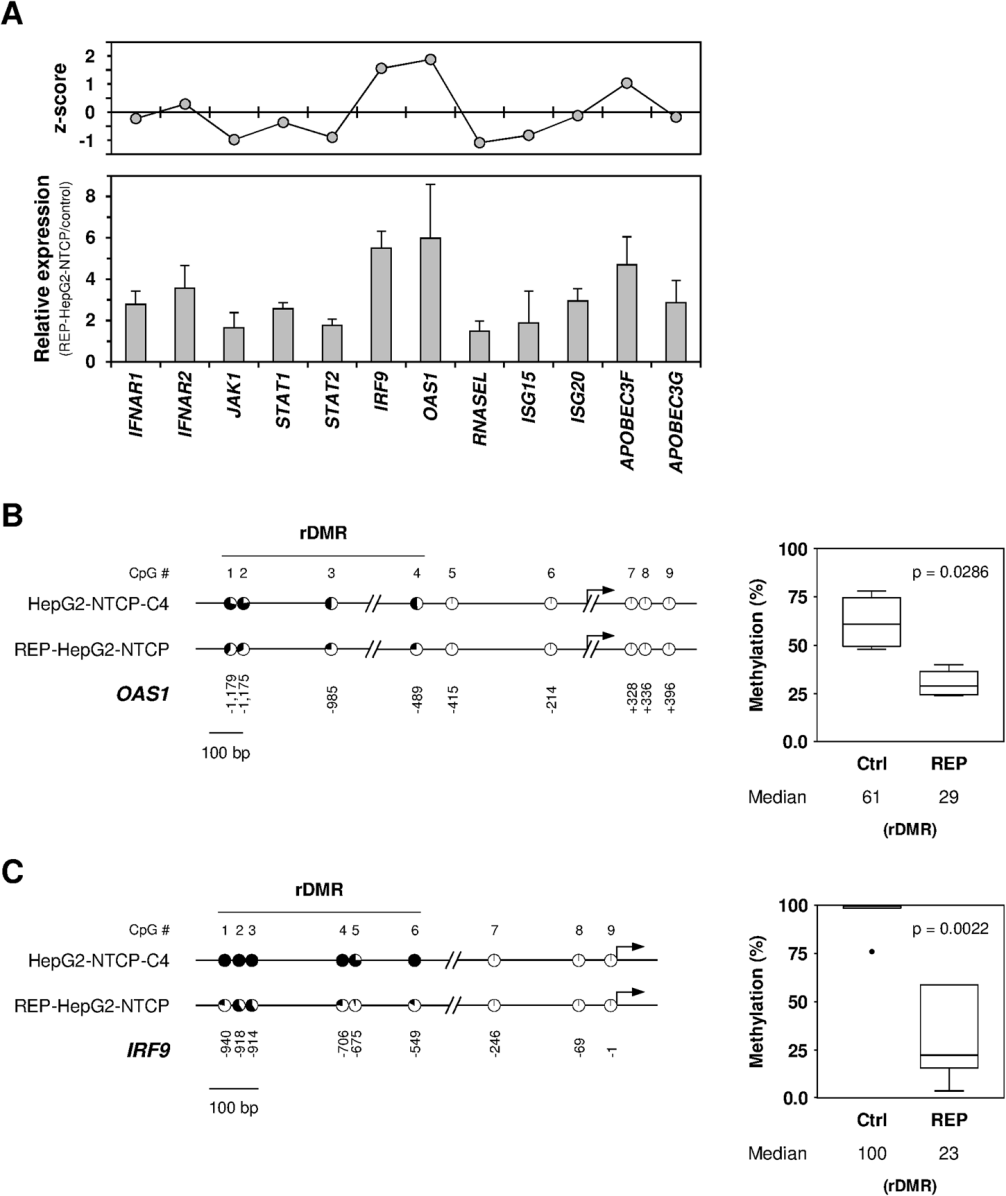
Analysis of *OAS1* and *IRF9* methylation after epigenetic reprogramming. **A** Relative expression levels of interferon-alpha and interferon-beta receptor subunit 1 and 2 (*IFNAR1* and *IFNAR2*), Janus kinase 1 (*JAK1*), signal transducer and activator of transcription 1 and 2 (*STAT1* and *STAT2*), ribonuclease L (*RNASEL*), ISG15 ubiquitin-like modifier (ISG15), *ISG20*, *APOBEC3F*, and *APOBEC3G*. The data show gene expression fold changes in the REP-HepG2-NTCP cells relative to non-reprogrammed control cells and *z* scores for each gene relative to all analyzed genes (*N* = 12). **B** Comparison of *OAS1* and **C** *IRF9* methylation profiles between REP-HepG2-NTCP cells and HepG2-NTCP-C4 cells. The COBRA data showed reprogramming DMRs (rDMRs) located from 549 to 940 bp and from 489 to 1179 bp upstream of the *OAS1* and *IRF9* TSSs, respectively. The black arrows indicate the *OAS1* and *IRF9* TSSs and the black circles represent the methylation percentage for each analyzed CpG site. The boxplots show the differential methylation levels of *OAS1*-rDMR and *IRF9*-rDMR in the REP-HepG2-NTCP cells (REP) compared with those of the control HepG2-NTCP-C4 cells (Ctrl). Significant differences in methylation levels were achieved at **p < 0.01 for *OAS1*-rDMR and *p < 0.05 for *IRF9*-rDMR (Mann–Whitney *U* test).

To clarify the molecular mechanism by which ISG induction was restored in epigenetically reprogrammed cells, we first analyzed the basal expression of several genes related to the antiviral response and specific genes that characterized the IFNα/JAK/STAT pathway. Our data showed that 5-AZA treatment led to a global augmentation in the expression of these markers without IFNα stimulation (Fig. 4A). z scores indicated that *IRF9* and *OAS1* were the most prominently amplified genes.

To determine whether the increased expression of *IRF9* and *OAS1* was the consequence of an epigenetic mechanism directly linked to the demethylating treatment during the reprogramming procedure, we analyzed the methylation profile of the genomic region upstream of the *IRF9* and *OAS1* transcription start site (TSS). A combined bisulfite restriction analysis (COBRA)^20^ showed that specific CpG sites associated with *OAS1* were differentially methylated between naive and reprogrammed HepG2-NTCP-C4 cells (Fig. 4B), delineating a differentially methylated region (DMR). The high degree of *OAS1*-DMR methylation measured before 5-AZA treatment (61% versus 29%, *p* = 0.0286, Mann–Whitney *U* test) was consistent with the epigenetic silencing of *OAS1* in the non-reprogrammed HepG2-NTCP-C4 cells. Another DMR was identified upstream of the *IRF9* TSS, which was also hypermethylated in HepG2-NTCP-C4 cells (Fig. 4C). Similar to its effect on *OAS1*-DMR, 5-AZA treatment promoted major demethylation of *IRF9*-DMR (23% versus 100%, *p* = 0.0022, Mann–Whitney *U* test), which was associated with the epigenetic unmasking of this gene. The methylation profiles of other critical ISGs, such as *ISG20* and *APOBEC3G*, were also determined by COBRA. However, the COBRA data showed no methylation in the CpG sites upstream of the *ISG20* or *APOBEC3G* TSS (S. Figure 2), suggesting that a mechanism independent of DNA methylation was responsible for the increased expression of these two genes in REP-HepG2-NTCP cells.

### Epigenetic reprogramming increases NTCP expression in HepG2-NTCP-C4 cells

**Fig. 5:**
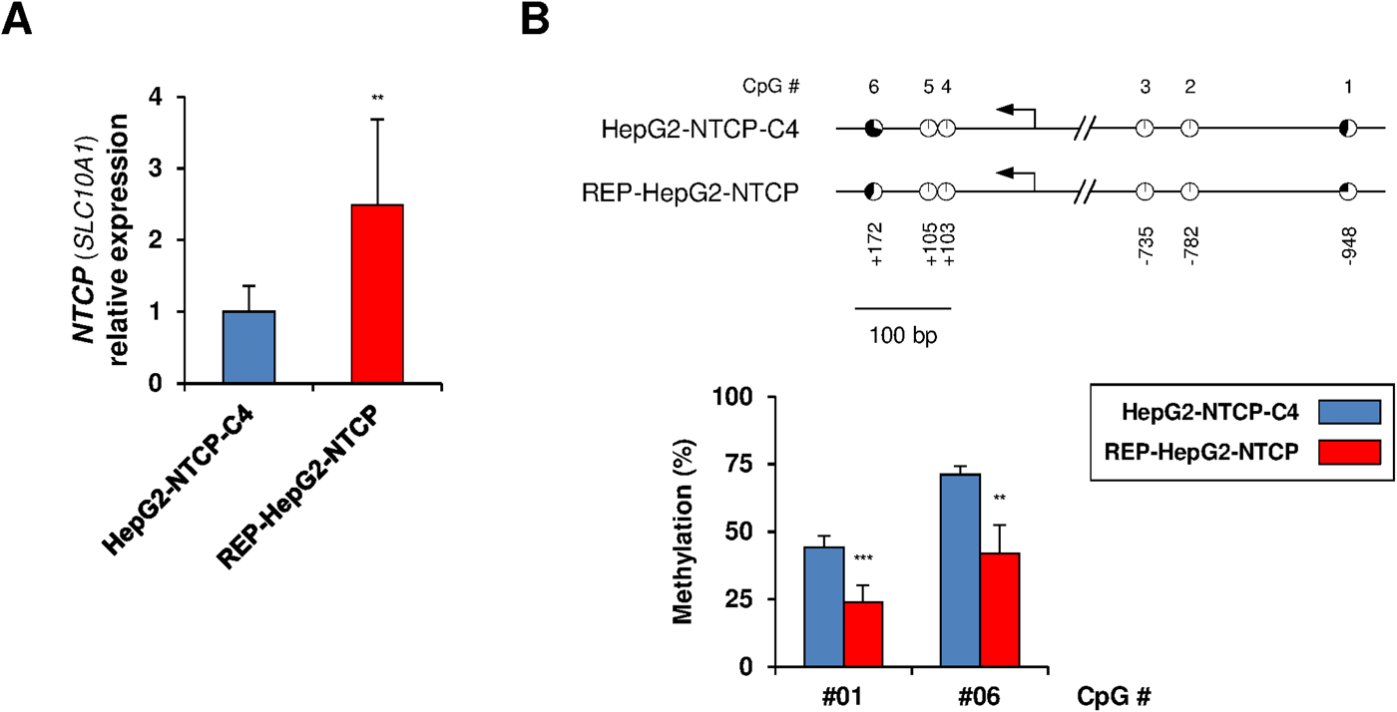
Expression and methylation levels of *NTCP* in REP-HepG2-NTCP cells. **A** Relative expression level of *NTCP* (*SLC10A1* gene) in REP-HepG2-NTCP cells. Non-reprogrammed HepG2-NTCP-C4 cells were used as controls. The histograms show the means ± SD. **p < 0.01 (*t* test). **B** Methylation status of the *NTCP* promoter region as determined by COBRA. Two CpG sites located 948 bp upstream and 172 bp downstream of the *NTCP* TSS were found to be differentially methylated between the REP-HepG2-NTCP cells and control HepG2-NTCP-C4 cells. A significant difference in CpG methylation levels was reached at *p* < 0.001 (CpG #01) and *p* < 0.01 (CpG #06) (*t* test).

HepG2-NTCP cell lines are characterized by stable overexpression of *NTCP*, which confers the susceptibility of these cells to HBV infection^12,13^. In line with our previous studies using the parental HepG2 cells^17^, we observed that *NTCP* expression was increased in REP-HepG2-NTCP cells compared with control HepG2-NTCP-C4 cells (Fig. 5A). Although no DMR was found in the *NTCP* promoter, two distinct CpG sites appeared differentially methylated in the control and reprogrammed cells (Fig. 5B). These CpG sites were located 948 bp upstream and 172 bp downstream of the *NTCP* TSS and exhibited methylation of 44% and 71%, respectively, in naive HepG2-NTCP-C4 cells, as determined by COBRA. The augmentation in *NTCP* levels was correlated with significant demethylation of these two CpG sites in REP-HepG2-NTCP cells, with methylation of 24% for CpG site #1 and 42% for CpG site #6 (*p* = 0.0003 and *p* = 0.0018, respectively, *t* test).

### CDM-3008 shows antiviral effects in REP-HepG2-NTCP cells

**Fig. 6:**
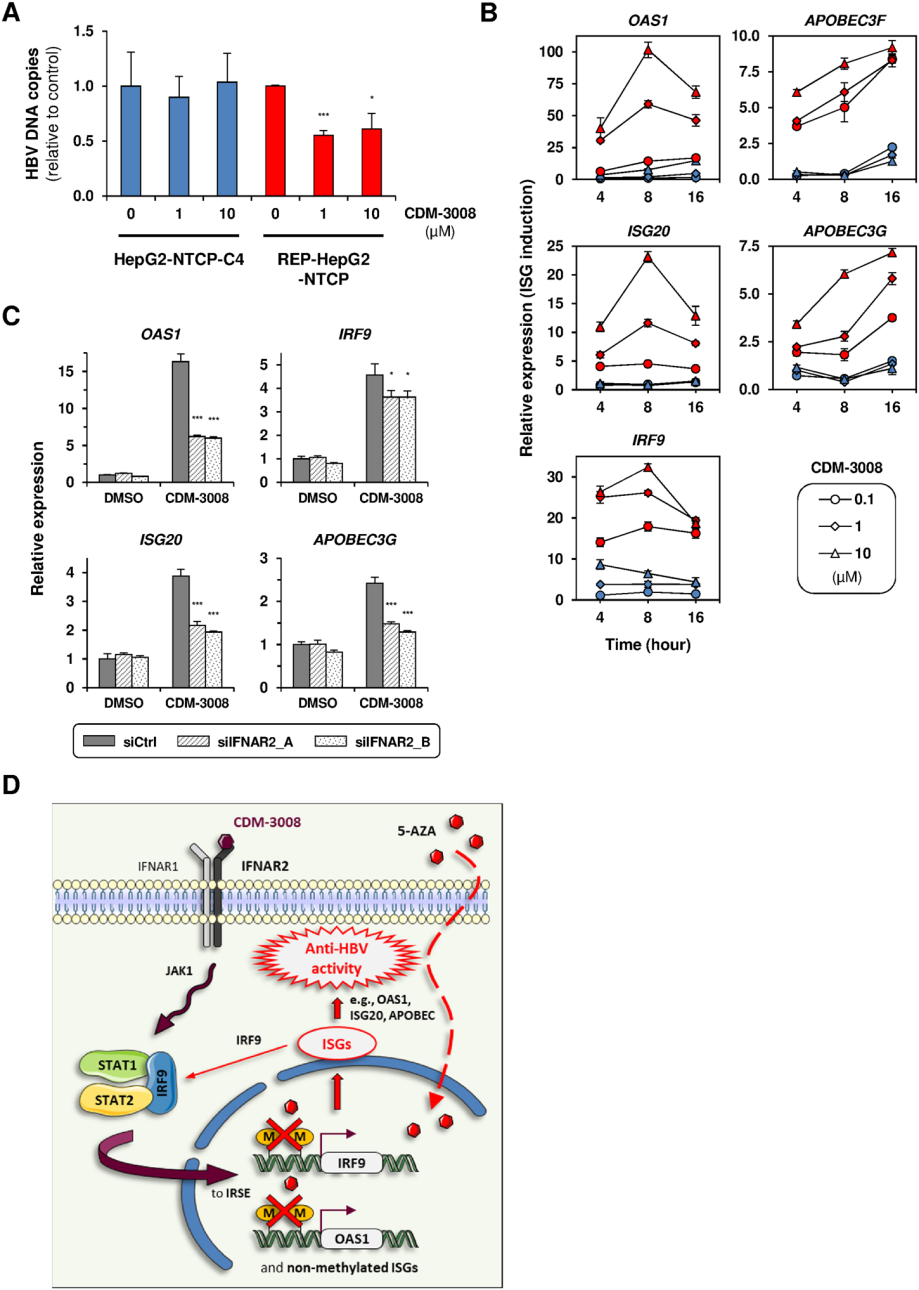
Assessment of CDM-3008 activity in REP-HepG2-NTCP cells. **A** Measurement of HBV DNA levels in HBV-infected cells after CDM-3008 treatment. Following epigenetic reprogramming (2 µM 5-AZA for 10 days) and HBV infection (MOI of 100 for 24 h), REP-HepG2-NTCP cells were treated with 1 and 10 µM CDM-3008 every 3 days. Total DNA was collected for analysis after 9 days of treatment. No cytotoxic effect was induced by the compound in cells after treatment (S. Figure 1). Statistically significant differences in HBV DNA levels relative to the level in the untreated cells were achieved at ***p < 0.001 and *p < 0.05 with 1 and 10 µM CDM-3008, respectively (*t* test). **B** Relative expression levels of *OAS1*, *ISG20*, *APOBEC3F*, *APOBEC3G*, and *IRF9* after CDM-3008 treatment at the indicated times and concentrations. The data from the REP-HepG2-NTCP and naive HepG2-NTCP-C4 cells are depicted in red and blue, respectively. **C** Relative expression of *OAS1*, *ISG20*, *APOBEC3G*, and *IRF9* after *IFNAR2* knockdown. Forty-eight hours after transfection with control siRNAs (siCtrl) or *IFNAR2* siRNAs (siIFNAR2_A and siIFNAR2_B), REP-HepG2-NTCP cells were treated with 1 µM CDM-3008 for 8 h. Significance related to siCtrl-transfected cells was evaluated by *t* test: *p < 0.05 and ***p < 0.001. All histograms depicted in the figure represent the means ± SD. **D** Proposed mechanism by which epigenetic reprogramming of HepG2-NTCP-C4 cells restores, in part, via *OAS1* and *IRF9* epigenetic unmasking, ISG induction and anti-HBV activity in response to CDM-3008 treatment.

To test the potential of epigenetic reprogramming for the evaluation of experimental IFN-like compounds, REP-HepG2-NTCP cells were exposed to CDM-3008, an IFN agonist previously characterized by high-throughput chemical library screening^21,22^. Our results showed that HBV DNA levels decreased by 44.58% ± 0.09% (*p* = 0.0002, *t* test) and 39.02% ± 0.07% (*p* = 0.0115, *t* test) in the infected REP-HepG2-NTCP cells when treated with 1 and 10 µM CDM-3008, respectively (Fig. 6A). Similar to IFNα, HBV DNA levels remained unchanged after CDM-3008 treatment in naive HepG2-NTCP-C4 cells. HBV DNA inhibition after CDM-3008 treatment was associated with the marked induction of ISGs in the REP-HepG2-NTCP cells, which was limited in non-reprogrammed cells (Fig. 6B). We confirmed that no cytotoxic effect was induced in cells after 5-AZA and CDM-3008 treatment (S. Figure 1B). Considering that CDM-3008 antiviral action is closely correlated to the levels of expression of cell-surface interferon-alpha and -beta receptor subunit 2 (*IFNAR2*)^21,23^, we analyzed ISG induction by CDM-3008 after knocking down *IFNAR2* using siRNAs. First, the efficiency of the siRNAs to inhibit *IFNAR2* specifically was validated (S. Figure 3). Next, our data showed that *IFNAR2* silencing jeopardized the induction of ISGs after CDM-3008 treatment in REP-HepG2-NTCP cells (Fig. 6C). For instance, *OAS1* expression was decreased by ~63% in the CDM-3008-stimulated REP-HepG2-NTCP cells after *IFNAR2* knockdown (*p* = 0.0003, *t* test). Combined, our data supported the adequacy of the REP-HepG2-NTCP cell system for use in the functional assessment of IFN-like compounds, such as CDM-3008 (Fig. 6D).

## Discussion

To establish an ideal cell culture-based system for HBV studies, consistent virus infection/replication rates and antiviral responses are required. This study provides evidence for efficient epigenetic reprogramming of the HBV-susceptible HepG2-NTCP-C4 clone toward IFNα and CDM-3008-responsive cells. We report that restoration of the antiviral properties of these molecules was associated with the epigenetic unmasking of key ISGs through DMR demethylation in REP-HepG2-NTCP cells.

Epigenetic processes are essential for orchestrating gene expression in eukaryotic organisms^24,25^. Cancer cells are usually characterized by aberrant hypermethylation patterns restricted to CpG-rich regions that lead to the silencing of critical genes and microRNAs associated with tumor suppression or cell differentiation^26–29^. Given the reversible nature of epigenetic modifications^30,31^, the use of demethylating drugs is promising for cell epigenetic reprogramming, as these compounds are unique tools for reversing the cancer phenotype and restoring tissue-specific gene expression. We previously reported the efficient reactivation of epigenetically silenced genes associated with cell differentiation through the use of a demethylating treatment in hepatic and pancreatic cancer cells^16–18^. In the present work, we connected the absence of an antiviral response in naive HepG2-NTCP-C4 cells with the epigenetic silencing of two major ISGs, *OAS1* and *IRF9*. In contrast, epigenetic reprogramming efficiently restored the IFN-stimulated induction of *OAS1* and *IRF9*. Recovery of antiviral activity in REP-HepG2-NTCP cells was also associated with the induction of additional ISGs, such as *ISG20* and *APOBEC3G*, independent of a DMR-mediated regulation mechanism. Our findings suggest that *ISG20* and *APOBEC3G* probably belong to a set of ISGs, which are not directly reactivated by the demethylating compound during epigenetic reprogramming. Thus, HepG2-NTCP-C4 cell reprogramming is most likely the consequence of a sequential reactivation of ISGs, characterized first (i) by the direct demethylation and re-expression of specific epigenetically controlled ISGs (e.g., *OAS1*) and key transcription factors, such as *IRF9* and (ii) by the subsequent initiation of the global transcription of ISGs (Fig. 6D).

As the main target of HBV infection^32^, human hepatocytes appear to be the most appropriate model for anti-HBV drug screening. However, access to PHHs is limited because of the scarcity of liver donors and the substantial price of this biological material. Furthermore, the inability to expand PHHs in vitro and the extreme variability of the antiviral response among donors limit their utilization. The HepaRG cell model is an interesting substitute for PHHs for use in HBV studies^9^, but these cells require several weeks of treatment with dimethyl sulfoxide (DMSO) to fully differentiate into a mixture of bile epithelial cells and hepatocytes^33^, which can be a source of bias for reliable antiviral response studies. A significant step forward has been made since the discovery of the HBV entry receptor NTCP and the development of various *NTCP*-overexpressing HCC cell models, such as HepG2-NTCP cells^12–14^. The HepG2-NTCP cell lines probably represent the most common and popular models for HBV studies because of the ease of access of these cells, their high transfection rate, and their consistent antiviral response inherent to their clonogenic nature. In an elegant study, Qiao and collaborators established various HepG2-NTCP clones that stably overexpressed *NTCP* at different levels and demonstrated that HBV permissiveness was tightly correlated with NTCP protein levels^34^. The authors also showed that HBV replication was significantly promoted by treating the HBV-infected HepG2-NTCP clones with DMSO and hydrocortisone. It would be interesting to apply the epigenetic reprogramming protocol to improve the antiviral activity in this optimized HBV cell culture model for potential drug-screening applications.

In conclusion, our study demonstrates that epigenetic reprogramming of HepG2-NTCP-C4 cells using 5-AZA promotes the anti-HBV action of IFNα. REP-HepG2-NTCP cells also appear appropriate for the functional and therapeutic assessment of IFN-like compounds. Additional investigation will be essential to determine the impact of epigenetic modifications on HBV genome and the value of epigenetically reprogrammed cells for future drug-screening applications. At last, it is certainly appealing to consider that epigenetic reprogramming could also be valuable for improving other cell-virus culture systems that lack appropriate cell-mediated antiviral responses for treatment evaluation.

## Materials and methods

### Human hepatic cell lines and reagents

HepG2-NTCP-C4 cells and HepG2.2.15.7 cells were obtained from the National Institute of Infectious Diseases (Tokyo, Japan). The HepG2-NTCP-C4 cells were maintained in a Dulbecco’s modified Eagle’s medium (DMEM)/F-12, GlutaMAX mixture (Gibco) supplemented with insulin (5 µg/mL; Wako), hydrocortisone (50 µM; Wako), 10 mM HEPES (Gibco), penicillin (100 IU/mL; Gibco), streptomycin (100 µg/mL; Gibco), and 10% fetal bovine serum (FBS; Gibco). The HepG2.2.15.7 cells were maintained in a DMEM/F-12, GlutaMAX mixture supplemented with insulin (5 µg/mL; Wako), penicillin (100 IU/mL), streptomycin (100 µg/mL), and 10% FBS. HepG2 cells were purchased from the American Type Culture Collection and maintained in DMEM (Gibco) supplemented with 2 mM l-glutamine (Gibco), penicillin (50 IU/mL), streptomycin (50 µg/mL), and 10% FBS. The demethylating compound 5-AZA (PubChem CID: 9444) was purchased from Sigma. 5-AZA was dissolved in phosphate-buffered saline (PBS) as a 10 mM stock solution, filtered (0.22 µM), and stored at −20 °C in aliquots. The 5-AZA-supplemented medium was prepared every day. IFNα solution was obtained from Sumitomo Dainippon Pharma. CDM-3008 (RO8191; PubChem CID: 2768133) was obtained from SIGMA and dissolved in DMSO as a 1,000 µM working solution before use.

### HBV production and cell infection

HBV particles were collected from HepG2.2.15.7 cells, which stably express and replicate the virus (genotype D). The culture medium containing viral particles was collected from confluent cells. After filtration (0.22 µM), the medium was incubated with a mixture of 23% NaCl and 50% PEG 8000 for at least 2 h at 4°C. Next, HBV particles were purified by ultracentrifugation, washed with PBS, resuspended in cell culture medium, and stored at −80°C until used for cell infection. REP-HepG2-NTCP cells and HepG2-NTCP-C4 cells were seeded in 96-well plates 24 h before infection. The cells were infected using a mix of 10 µL 40% PEG 8000 and 90 µL cell culture medium (without 5-AZA) containing HBV particles at an MOI of 100 for 24 h.

### Cell transfection

REP-HepG2-NTCP cells were seeded at a density of 200,000 cells/cm^2^ in 35-mm-diameter culture dishes and transfected the next day using TransFectin lipid reagent (Bio-Rad). Cells were incubated with a transfection mix containing 100 nmol/L siRNA and 5 µL of TransFectin in a 1.2 mL total volume of serum- and antibiotic-free Opti-MEM (Invitrogen) for 5 h. Human *IFNAR2* siRNAs (IDs s7185 and s7186 for siIFNAR2_A and siIFNAR2_B, respectively) (Ref. #4390824) and Silencer Select Negative Control #1 siRNA (siCtrl; Ref. #4390843) were purchased from Ambion.

### Total DNA and RNA isolation

Total DNA was purified from REP-HepG2-NTCP cells and naive HepG2-NTCP-C4 cells using an Agencourt DNAdvance Genomic DNA Isolation kit and a Biomek NXp automatic dispenser from Beckman Coulter according to the manufacturer’s instructions. Total RNA was purified using an Agencourt RNAdvance Cell v2 and a Biomek NXp automatic from Beckman Coulter according to the manufacturer’s instructions. Before elution, the RNA samples were treated with five units DNase I (Nippon Gene) at 25°C for 15 min. For the microarray analysis, total RNA was purified from HepG2 cells using a miRNeasy Mini kit (Qiagen) according to the manufacturer’s recommendations, and samples were treated with 1 mL of DNase at 37°C for 30 min using a TURBO DNA-free kit (Ambion). DNA and RNA sample concentrations were determined using a DeNovix spectrophotometer.

### Gene expression microarray and GSEA

After purification, RNA samples from 5-AZA-treated HepG2 cells (2.5 µM for 10 days) were subjected to a gene expression microarray. Untreated HepG2 cells were used as controls. RNA labeling and hybridization were performed using an Agilent one-color low RNA input linear amplification kit labeling protocol and an Agilent one-color gene expression hyb/wash protocol (Agilent Technologies). The microarray slides were scanned in an Agilent G2505C Microarray Scanner at 3-micron resolution (Agilent Technologies). The raw data were processed using Agilent’s Feature Extraction Software version 10.7.3.1 to analyze the array and calculate the intensities of the measured spots. The Agilent Gene Expression Platform was Agilent-039494. GSEA was performed using the HALLMARK gene datasets (http://software.broadinstitute.org/gsea).

### mRNA and HBV DNA real-time quantitative PCR

To evaluate gene expression levels, cDNAs were first synthesized from 1 µg of purified RNA using PrimeScript RT Master Mix (TaKaRa) according to the manufacturer’s recommendations. Real-time quantitative PCR was performed using TB Green Premix Ex Taq II (TaKaRa) and a CFX96 Real-Time PCR Detection System (Bio-Rad). After initial denaturation at 95°C for 30 sec, the thermal cycles were repeated 40 times as follows: 95°C for 5 sec and 60°C for 30 sec. The housekeeping gene glyceraldehyde 3-phosphatase dehydrogenase (*GAPDH*) was used to normalize the cDNA levels. The sequences of the human primers used for gene amplification are shown in **S**. Table 2. HBV DNA levels were determined by real-time quantitative PCR using Probe qPCR mix (TaKaRa) from 50 ng of total DNA. The PCR conditions were 95°C for 1 min, followed by 50 cycles of 95°C for 10 sec and 60°C for 30 sec. The HBV DNA TaqMan probe sequence was 5′-[FAM] TATCGCTGGATGTGTCTGCGGCGT[TAM]-3′. The sequences of the specific primers used for HBV DNA detection were 5′-ACTCACCAACCTCCTGTCCT-3′ (forward primer) and 5′-GACAAACGGGCAACATACCT-3′ (reverse primer).

### DNA methylation analysis

COBRA was used to assess the methylation status of *OAS1*, *IRF9*, *ISG20*, *APOBEC3G*, and *NTCP* before and after epigenetic reprogramming of HepG2-NTCP-C4 cells. An in silico analysis using the UCSC Genome Bioinformatics tool (http://genome.ucsc.edu) was performed to identify the CpG sites located upstream of the TSS of these genes. MethPrimer (http://www.urogene.org/methprimer) was used to design the COBRA primers required to amplify the genomic regions containing the CpGs of interest (S. Table 3). One microgram of genomic DNA was subjected to bisulfite conversion treatment using an EpiTect Plus kit (QIAGEN). Next, COBRA PCR was performed as follows: after an initial denaturation step of 94°C for 3 min, the following thermal cycles were repeated 40 times: 94°C for 10 sec, 55°C for 50 sec, and 72°C for 1 min. Each COBRA PCR was performed in a total volume of 10 µL, which contained 0.5 units of Hot Start Taq polymerase (TaKaRa), 10 pmol of primers, and 1 µL of bisulfite-treated DNA. After PCR amplification, COBRA PCR products were digested with three units of restriction enzyme. Finally, the restriction products were separated by polyacrylamide gel electrophoresis and visualized by ethidium bromide staining. The bands were densitometrically analyzed using ImageJ software (v1.50i, National Institutes of Health, USA) to quantify the unmethylated (*U*) and methylated (*M*) restriction fragments. The methylation levels were calculated for each locus using the formula (*M*×100)/(*M* + *U*); they are expressed as methylation percentages.

### Cell viability assay

For the evaluation of the time- and dose-dependent cytotoxicity of 5-AZA, HepG2-NTCP-C4 cells were seeded at 15,000 cells/well in 96-well plates and treated with 5-AZA at the indicated concentrations. The 5-AZA-supplemented medium was changed every 2 days. Cell viability was determined at the indicated times using a Cell Proliferation Kit II from Roche, according to the manufacturer’s instructions (XTT assay). Absorbance at 492 nm and 690 nm was measured using an EnSight Multimode Plate Reader (PerkinElmer). REP-HepG2-NTCP cell and HepG2-NTCP-C4 cell viabilities were determined after IFNα or CDM-3008 treatment using the same method.

### Statistical analysis

All experimental data are presented as the means ± SD. Student’s *t* test was performed to estimate statistical significance. The equality of the variances was tested using an *F* test, and a correction was performed in cases of unequal variance (Welch *t* test). All *p* values were two-tailed. The statistical significance of differences in *OAS1*-DMR and *IRF9*-DMR methylation levels between reprogrammed and control cells was assessed using a Mann–Whitney *U* test. All depicted data are representative of at least three independent experiments.

## Supplementary information

S. Table 1

S. Table 2

S. Table 3

aj-checklist

Author contribution

S. Figure 1

S. Figure 2

S. Figure 3

SUPPLEMENTARY FIGURE LEGENDS

## Data Availability

Microarray data are deposited in the NCBI Gene Expression Omnibus (GEO) under the accession number GSE160648.
