# Supplementary material for: Epigenetic reprogramming promotes the antiviral action of IFNα in HBV-infected cells
Source: Cell Death Discov. 2021 Jun 2;7:130. doi: 10.1038/s41420-021-00515-y (PMC8170866; doi:10.1038/s41420-021-00515-y)
Supplement: Supplementary file 1 — S. Table 1 [file 41420_2021_515_MOESM1_ESM.docx]

**S. Table 1.** List of the genes involved in the interferon-alpha response and enriched in the reprogrammed HepG2 cells (versus untreated HepG2 cells)

| **GENE** | **DESCRIPTION** | **RANK IN GENE LIST** | **RANK METRIC SCORE** | **RUNNING ES** | **CORE ENRICHMENT** |
| --- | --- | --- | --- | --- | --- |
| **PARP9** | poly(ADP-ribose) polymerase family member 9 | 96 | 5.000 | 0.024 | **Yes** |
| **NMI** | N-myc and STAT interactor | 117 | 5.000 | 0.051 | **Yes** |
| **IRF7** | interferon regulatory factor 7 | 259 | 5.000 | 0.072 | **Yes** |
| **C1S** | complement C1s | 561 | 4.781 | 0.085 | **Yes** |
| **LAMP3** | lysosomal associated membrane protein 3 | 613 | 4.663 | 0.109 | **Yes** |
| **PARP12** | poly(ADP-ribose) polymerase family member 12 | 656 | 4.582 | 0.132 | **Yes** |
| **PROCR** | protein C receptor | 823 | 4.287 | 0.149 | **Yes** |
| **IRF9** | interferon regulatory factor 9 | 968 | 4.016 | 0.164 | **Yes** |
| **CASP1** | caspase 1 | 1098 | 3.779 | 0.180 | **Yes** |
| **PARP14** | poly(ADP-ribose) polymerase family member 14 | 1227 | 3.563 | 0.194 | **Yes** |
| **TRAFD1** | TRAF-type zinc finger domain containing 1 | 1289 | 3.469 | 0.210 | **Yes** |
| **NUB1** | negative regulator of ubiquitin like proteins 1 | 1291 | 3.468 | 0.230 | **Yes** |
| **UBA7** | ubiquitin like modifier activating enzyme 7 | 1360 | 3.363 | 0.245 | **Yes** |
| **DHX58** | DExH-box helicase 58 | 1408 | 3.283 | 0.262 | **Yes** |
| **HELZ2** | helicase with zinc finger 2 | 1417 | 3.276 | 0.280 | **Yes** |
| **TDRD7** | tudor domain containing 7 | 1800 | 2.822 | 0.277 | **Yes** |
| **RTP4** | receptor transporter protein 4 | 2022 | 2.583 | 0.282 | **Yes** |
| **IFIT2** | interferon induced protein with tetratricopeptide repeats 2 | 2229 | 2.397 | 0.285 | **Yes** |
| **CD47** | CD47 molecule | 2288 | 2.346 | 0.296 | **Yes** |
| **IRF1** | interferon regulatory factor 1 | 2289 | 2.346 | 0.309 | **Yes** |
| **ISG20** | interferon stimulated exonuclease gene 20 | 2379 | 2.262 | 0.317 | **Yes** |
| **IFI27** | interferon alpha inducible protein 27 | 2492 | 2.172 | 0.324 | **Yes** |
| **CMPK2** | cytidine/uridine monophosphate kinase 2 | 2510 | 2.160 | 0.336 | **Yes** |
| **SLC25A28** | solute carrier family 25 member 28 | 2571 | 2.119 | 0.345 | **Yes** |
| **MOV10** | Mov10 RISC complex RNA helicase | 2653 | 2.049 | 0.353 | **Yes** |
| **TXNIP** | thioredoxin interacting protein | 2714 | 1.995 | 0.361 | **Yes** |
| **PLSCR1** | phospholipid scramblase 1 | 2786 | 1.939 | 0.368 | **Yes** |
| **OAS1** | 2'-5'-oligoadenylate synthetase 1 | 3097 | 1.714 | 0.363 | **Yes** |
| **TAP1** | transporter 1, ATP binding cassette subfamily B member | 3182 | 1.667 | 0.369 | **Yes** |
| **PSMB8** | proteasome 20S subunit beta 8 | 3212 | 1.648 | 0.377 | **Yes** |
| **B2M** | beta-2-microglobulin | 3225 | 1.637 | 0.385 | **Yes** |
| **TRIM21** | tripartite motif containing 21 | 3470 | 1.475 | 0.382 | **Yes** |
| **CMTR1** | cap methyltransferase 1 | 3526 | 1.433 | 0.388 | **Yes** |
| **GBP2** | guanylate binding protein 2 | 3575 | 1.402 | 0.393 | **Yes** |
| **STAT2** | signal transducer and activator of transcription 2 | 3743 | 1.299 | 0.393 | **Yes** |
| **MX1** | MX dynamin like GTPase 1 | 3822 | 1.246 | 0.396 | **Yes** |
| **TMEM140** | transmembrane protein 140 | 3867 | 1.223 | 0.401 | **Yes** |
| **PSMB9** | proteasome 20S subunit beta 9 | 3940 | 1.184 | 0.404 | **Yes** |
| **CD74** | CD74 molecule | 4242 | 1.017 | 0.395 | **No** |
| **DDX60** | DExD/H-box helicase 60 | 4248 | 1.016 | 0.401 | **No** |
| **SP110** | SP110 nuclear body protein | 5530 | 0.635 | 0.344 | **No** |
| **GMPR** | guanosine monophosphate reductase | 5565 | 0.628 | 0.346 | **No** |
| **RSAD2** | radical S-adenosyl methionine domain containing 2 | 5572 | 0.626 | 0.349 | **No** |
| **OASL** | 2'-5'-oligoadenylate synthetase like | 5646 | 0.611 | 0.349 | **No** |
| **IFI35** | interferon induced protein 35 | 5651 | 0.610 | 0.352 | **No** |
| **IL7** | interleukin 7 | 5820 | 0.575 | 0.348 | **No** |
| **IFI44L** | interferon induced protein 44 like | 5900 | 0.556 | 0.347 | **No** |
| **UBE2L6** | ubiquitin conjugating enzyme E2 L6 | 5936 | 0.545 | 0.348 | **No** |
| **HERC6** | HECT and RLD domain containing E3 ubiquitin protein ligase family member 6 | 6229 | 0.475 | 0.337 | **No** |
| **CASP8** | caspase 8 | 6515 | 0.402 | 0.326 | **No** |
| **USP18** | ubiquitin specific peptidase 18 | 6944 | 0.293 | 0.308 | **No** |
| **CXCL10** | C-X-C motif chemokine ligand 10 | 6948 | 0.293 | 0.309 | **No** |
| **SAMD9** | sterile alpha motif domain containing 9 | 7603 | 0.138 | 0.279 | **No** |
| **BST2** | bone marrow stromal cell antigen 2 | 7924 | 0.052 | 0.264 | **No** |
| **ISG15** | ISG15 ubiquitin like modifier | 8045 | 0.023 | 0.259 | **No** |
| **ADAR** | adenosine deaminase RNA specific | 8104 | 0.011 | 0.256 | **No** |
| **IFIT3** | interferon induced protein with tetratricopeptide repeats 3 | 8572 | -0.087 | 0.234 | **No** |
| **CSF1** | colony stimulating factor 1 | 10039 | -0.265 | 0.166 | **No** |
| **IFI44** | interferon induced protein 44 | 10492 | -0.292 | 0.147 | **No** |
| **CCRL2** | C-C motif chemokine receptor like 2 | 11096 | -0.338 | 0.120 | **No** |
| **SAMD9L** | sterile alpha motif domain containing 9 like | 11432 | -0.365 | 0.106 | **No** |
| **PSME1** | proteasome activator subunit 1 | 12812 | -0.574 | 0.044 | **No** |
| **EPSTI1** | epithelial stromal interaction 1 | 12892 | -0.585 | 0.044 | **No** |
| **ELF1** | E74 like ETS transcription factor 1 | 13618 | -0.681 | 0.014 | **No** |
| **TRIM5** | tripartite motif containing 5 | 13810 | -0.712 | 0.009 | **No** |
| **TRIM25** | tripartite motif containing 25 | 13921 | -0.739 | 0.007 | **No** |
| **OGFR** | opioid growth factor receptor | 15349 | -1.172 | -0.053 | **No** |
| **IL4R** | interleukin 4 receptor | 16016 | -1.398 | -0.077 | **No** |
| **LGALS3BP** | galectin 3 binding protein | 16642 | -1.633 | -0.097 | **No** |
| **LPAR6** | lysophosphatidic acid receptor 6 | 16835 | -1.715 | -0.097 | **No** |
| **EIF2AK2** | eukaryotic translation initiation factor 2 alpha kinase 2 | 16902 | -1.745 | -0.090 | **No** |
| **RNF31** | ring finger protein 31 | 17139 | -1.837 | -0.091 | **No** |
| **WARS1** | tryptophanyl-tRNA synthetase 1 | 17158 | -1.843 | -0.081 | **No** |
| **MVB12A** | multivesicular body subunit 12A | 17217 | -1.864 | -0.074 | **No** |
| **TRIM14** | tripartite motif containing 14 | 17322 | -1.914 | -0.068 | **No** |
| **LY6E** | lymphocyte antigen 6 family member E | 17448 | -1.968 | -0.063 | **No** |
| **TRIM26** | tripartite motif containing 26 | 17592 | -2.029 | -0.058 | **No** |
| **NCOA7** | nuclear receptor coactivator 7 | 17624 | -2.041 | -0.048 | **No** |
| **CNP** | 2',3'-cyclic nucleotide 3' phosphodiesterase | 17770 | -2.114 | -0.043 | **No** |
| **GBP4** | guanylate binding protein 4 | 18023 | -2.235 | -0.042 | **No** |
| **BATF2** | basic leucine zipper ATF-like transcription factor 2 | 18046 | -2.245 | -0.031 | **No** |
| **IFITM3** | interferon induced transmembrane protein 3 | 18147 | -2.296 | -0.023 | **No** |
| **TENT5A** | terminal nucleotidyltransferase 5A | 18678 | -2.612 | -0.033 | **No** |
| **LAP3** | leucine aminopeptidase 3 | 19057 | -2.860 | -0.035 | **No** |
| **PSME2** | proteasome activator subunit 2 | 19484 | -3.200 | -0.037 | **No** |
| **IFITM1** | interferon induced transmembrane protein 1 | 19985 | -3.678 | -0.040 | **No** |
| **PSMA3** | proteasome 20S subunit alpha 3 | 20283 | -4.026 | -0.031 | **No** |
| **RIPK2** | receptor interacting serine/threonine kinase 2 | 20447 | -4.266 | -0.015 | **No** |
| **PNPT1** | polyribonucleotide nucleotidyltransferase 1 | 20652 | -4.589 | 0.001 | **No** |
| **SELL** | selectin L | 20736 | -4.764 | 0.024 | **No** |

**GSEA analysis:** _HALLMARK_INTERFERON_ALPHA_RESPONSE
