# Supplementary material for: Epigenetic reprogramming promotes the antiviral action of IFNα in HBV-infected cells
Source: Cell Death Discov. 2021 Jun 2;7:130. doi: 10.1038/s41420-021-00515-y (PMC8170866; doi:10.1038/s41420-021-00515-y)
Supplement: Supplementary file 2 — S. Table 2 [file 41420_2021_515_MOESM2_ESM.docx]

**S. Table 2.** List of the primers used for mRNA real-time quantitative PCR

|  |  | |  |  |
| --- | --- | --- | --- | --- |
| Gene | Name | Sense | | Antisense |
|  |  |  | |  |
|  |  |  | |  |
| ***GAPDH***  ***IFNAR1***  ***IFNAR2***  ***JAK1***  ***STAT1***  ***STAT2***  ***IRF9***  ***OAS1***  ***RNASEL***  ***ISG15***  ***ISG20***  ***APOBEC3F*** | Glyceraldehyde 3-phosphatase dehydrogenase  Interferon alpha and beta receptor subunit 1  Interferon alpha and beta receptor subunit 2  Janus kinase 1  Signal transducer and activator of transcription 1  Signal transducer and activator of transcription 2  Interferon regulatory factor 9  2'-5'-oligoadenylate synthetase 1  Ribonuclease L  ISG15 ubiquitin like modifier  Interferon stimulated exonuclease gene 20  Apolipoprotein B mRNA editing enzyme catalytic subunit 3F | aatcccatcaccatcttcca  atcggtgctccaaaacagtc  cgaaatttccggtccatct  tgtaaggagctggctgacct  ccgttttcatgacctcctgt  agctgctgaaggagctgaag  aggtccagctgtctggaaga  gccattgacatcatctgtgg  gccgctgtgtatggtaaggt  tgtcggtgtcagagctgaag  cttccaggcactgaaagagg  ctggctgtgctacgaagtga | | ttcacacccatgacgaacat  gtgctctggctttcacacaa  tcgtgtgtgcttctccactc  cacctgctcccctgtattgt  tgaatattccccgactgagc  ggctgggtttctaccacaaa  atggcatcctcttcctcctt  ggcttccagctgtctcctaac  atggatcaaggcatttctgc  gcccttgttattcctcacca  atcttccaccgagctgtgtc  agggggtccaggatacaaac |
| ***APOBEC3G***  ***SLC10A1*** | Apolipoprotein B mRNA editing enzyme catalytic subunit 3G  Solute carrier family 10 member 1 (*NTCP*) | tccacccacattcactttca  gggacatgaacctcagcatt | | ttccaaaagggaatcacgtc  cgtttggatttgaggacgat |
|  |  |  | |  |

The primers were designed using Primer3 v.0.4.0 (http://bioinfo.ut.ee/primer3-0.4.0/primer3).
