# Supplementary material for: Epigenetic reprogramming promotes the antiviral action of IFNα in HBV-infected cells
Source: Cell Death Discov. 2021 Jun 2;7:130. doi: 10.1038/s41420-021-00515-y (PMC8170866; doi:10.1038/s41420-021-00515-y)
Supplement: Supplementary file 3 — S. Table 3 [file 41420_2021_515_MOESM3_ESM.docx]

**S. Table 3.** Human primers used for COBRA

|  | |  |  |  |  |  |
| --- | --- | --- | --- | --- | --- | --- |
| Target | Sense | | Antisense | CpG # |  |  |
|  |  | |  |  |  |  |
|  |  | |  |  |  |  |
| ***IRF9*** (01) | TATTTTGTATGAGGTGGAAGAGTTG | | CAAACACAATCAACACCAAATAACT | 1 to 3 |  |  |
| ***IRF9*** (02) | GGTGTTGATTGTGTTTGAGTTTTAG | | ATTACAAAAAACCCTTTTCAACAAA | 4 to 6 |  |  |
| ***IRF9*** (03) | ATTGTGGGGGAATGTAGATGTATAG | | AAAAAAAACCTTTTCCCAAAAATC | 7 and 8 |  |  |
| ***IRF9*** (04)  ***OAS1*** (01)  ***OAS1*** (02)  ***OAS1*** (03)  ***OAS1*** (04)  ***OAS1*** (05)  ***SLC10A1*** (01)  ***SLC10A1*** (02)  ***SLC10A1*** (03)  ***ISG20*** (01)  ***ISG20*** (02)  ***APOBEC3G*** (01)  ***APOBEC3G*** (02) | TTAGATGTTGTTGTTTTTTAGTGGT  TGTAGGGTAGGGGAGTTTTAAAATT  AGGAGAGAGAGTTTATGGAATTTTTT  GGAGGATTTTTTGAATTTAGGAAGT  AAAAATTTGGAAAGTTTTATATTAAAA  TTGGGAGGTAGTTTTGTTGTTATTT  GTAATTTGAGAGAATTTTGGTTGTG  TTGGGGAAATGTTTGTTTTATTTT  TGAAGAATAATATGAATATTAGGATGA  TGGGATATTTTTTGAGTTTTAGTTTT  GTAAAGGAGGGGTATTTTGATATG  GGTTTATATTTATTTGTTGTTTTTTTT  GTAGGGAGGTTTTAGATAGGTTGGT | | CCAAACTAACTCCCATCCTTAACTA  ATTCCCTAACAATAACCCAATTACC  CTACCCCAACCTCCTAAATACCTA  CAAACACAAATCTCTTTTCTTTTTATTAAA  TATCCTTTAACCAACAACAAACAAC  CCTAAACTCACCTTTACCACCTTAA  CCCTTATCCCACCTCTACAATAAA  AATCTCCACAACCAAAATTCTCTC  AAAAAACATCCAACAAAAACTACAC  CATATCAAAATACCCCTCCTTTAC  CTCAACTACTACAACTACTCTCAAC  ACTAAAACCTCCTCTCCACCATC  AAATATAAACCTCAAATATCCCACC | 9  1 to 3  4  5  6  7 to 9  1 and 2  3  4 to 6  1 and 2  3 to 7  1  2 to 8 |  |  |
|  |  | |  |  |  |  |

All primers were designed using the UCSC Genome Bioinformatics Site (http://genome.ucsc.edu) and MethPrimer (http://www.urogene.org/methprimer).
