# Supplementary material for: Epigenetic reprogramming promotes the antiviral action of IFNα in HBV-infected cells
Source: Cell Death Discov. 2021 Jun 2;7:130. doi: 10.1038/s41420-021-00515-y (PMC8170866; doi:10.1038/s41420-021-00515-y)
Supplement: Supplementary file 5 — Author contribution [file 41420_2021_515_MOESM5_ESM.pdf]

DECLARATION OF CONTRIBUTIONS TO ARTICLE **ADMC**

Manuscript Number: CDDISCOVERY-21-1590-T

Journal Name: *Cell Death Discovery* (the 'Journal')

Proposed Title of the Contribution: Epigenetic reprogramming promotes the antiviral action of IFNα in HBV-infected cells (the 'Contribution)

Author(s): Luc Gailhouste, Masayuki Sudoh, Xian-Yang Qin, Koichi Watashi, Takaji Wakita, Takahiro Ochiya, Tomokazu Matsuura, Soichi Kojima, and Yutaka Furutani (the 'Authors')

For all *CDDiscovery* articles, each person named as an author in the published version must be able to show he or she has contributed substantially to the article.

Authorship credit should be based on 1) substantial contributions to conception and design, acquisition of data, or analysis and interpretation of data; 2) drafting the article or revising it critically for important intellectual content; and 3) final approval of the version to be published. Authors should meet conditions 1, 2 and 3.

Any person who cannot be shown to have made a substantial contribution to the article cannot be listed as an author in the final version. The name of any person who is deemed to have made a minor contribution can, however, appear in the Acknowledgments section of the article.

Please complete the table below to indicate the contributions of all named authors to the manuscript.

Author Full Name:	Specification of Contribution to the Manuscript:
Luc Gailhouste	condition 1, 2, and 3
Masayuki Sudoh	condition 1, 2, and 3
Xian-Yang Qin	condition 1, 2, and 3
Koichi Watashi	condition 1, 2, and 3
Takaji Wakita	condition 1, 2, and 3
Takahiro Ochiya	condition 1, 2, and 3
Tomokazu Matsuura	condition 1, 2, and 3
Soichi Kojima	condition 1, 2, and 3
Yutaka Furutani	condition 1, 2, and 3

Please complete the table below to indicate the contributions of all named authors to the figures.

Figure 1:

Luc Gailhouste, Masayuki Sudoh, Xian-Yang Qin, Koichi Watashi, Takaji Wakita, Takahiro Ochiya, Tomokazu Matsuura, Soichi Kojima, and Yutaka Furutani contributed to the acquisition of the presented data, or their analysis and interpretation. All authors approved the figure.

Figure 2:

Luc Gailhouste, Masayuki Sudoh, Xian-Yang Qin, Koichi Watashi, Takaji Wakita, Takahiro Ochiya, Tomokazu Matsuura, Soichi Kojima, and Yutaka Furutani contributed to the acquisition of the presented data, or their analysis and interpretation. All authors approved the figure.

Figure 3:

Luc Gailhouste, Masayuki Sudoh, Xian-Yang Qin, Koichi Watashi, Takaji Wakita, Takahiro Ochiya, Tomokazu Matsuura, Soichi Kojima, and Yutaka Furutani contributed to the acquisition of the presented data, or their analysis and interpretation. All authors approved the figure.

Figure 4:

Luc Gailhouste, Masayuki Sudoh, Xian-Yang Qin, Koichi Watashi, Takaji Wakita, Takahiro Ochiya, Tomokazu Matsuura, Soichi Kojima, and Yutaka Furutani contributed to the acquisition of the presented data, or their analysis and interpretation. All authors approved the figure.

Figure 5:

Luc Gailhouste, Masayuki Sudoh, Xian-Yang Qin, Koichi Watashi, Takaji Wakita, Takahiro Ochiya, Tomokazu Matsuura, Soichi Kojima, and Yutaka Furutani contributed to the acquisition of the presented data, or their analysis and interpretation. All authors approved the figure.

Figure 6:

Luc Gailhouste, Masayuki Sudoh, Xian-Yang Qin, Koichi Watashi, Takaji Wakita, Takahiro Ochiya, Tomokazu Matsuura, Soichi Kojima, and Yutaka Furutani contributed to the acquisition of the presented data, or their analysis and interpretation. All authors approved the figure.

Signed for and on behalf of the Author(s):

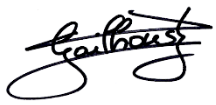

Print Name: Luc Gailhouste

Date: 2021-04-14
