# Supplementary figures and images for: Epigenetic reprogramming promotes the antiviral action of IFNα in HBV-infected cells
Source: Cell Death Discov. 2021 Jun 2;7:130. doi: 10.1038/s41420-021-00515-y (PMC8170866; doi:10.1038/s41420-021-00515-y)

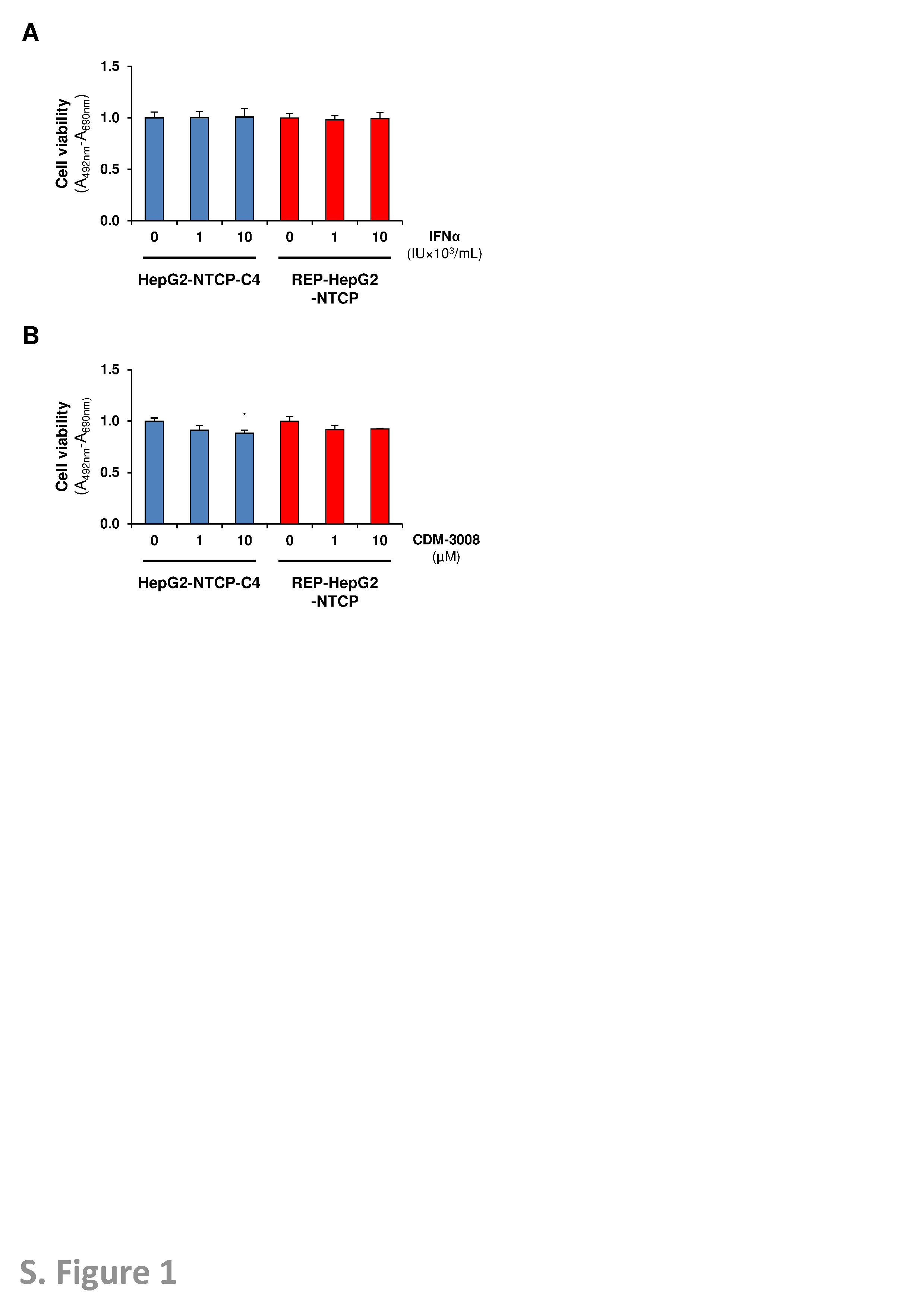

Supplement: Supplementary file 6 — S. Figure 1 [file 41420_2021_515_MOESM6_ESM.tif]

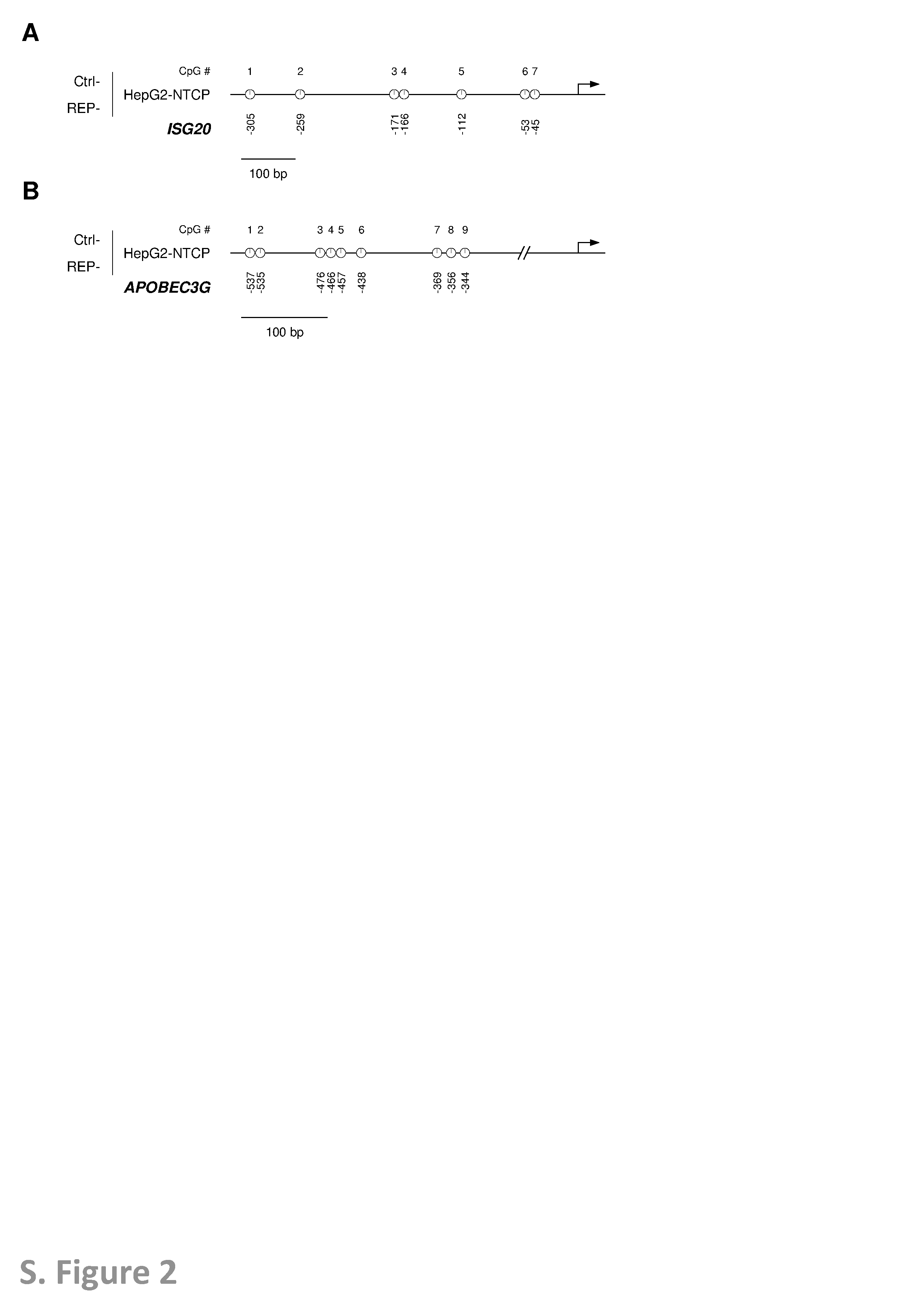

Supplement: Supplementary file 7 — S. Figure 2 [file 41420_2021_515_MOESM7_ESM.tif]

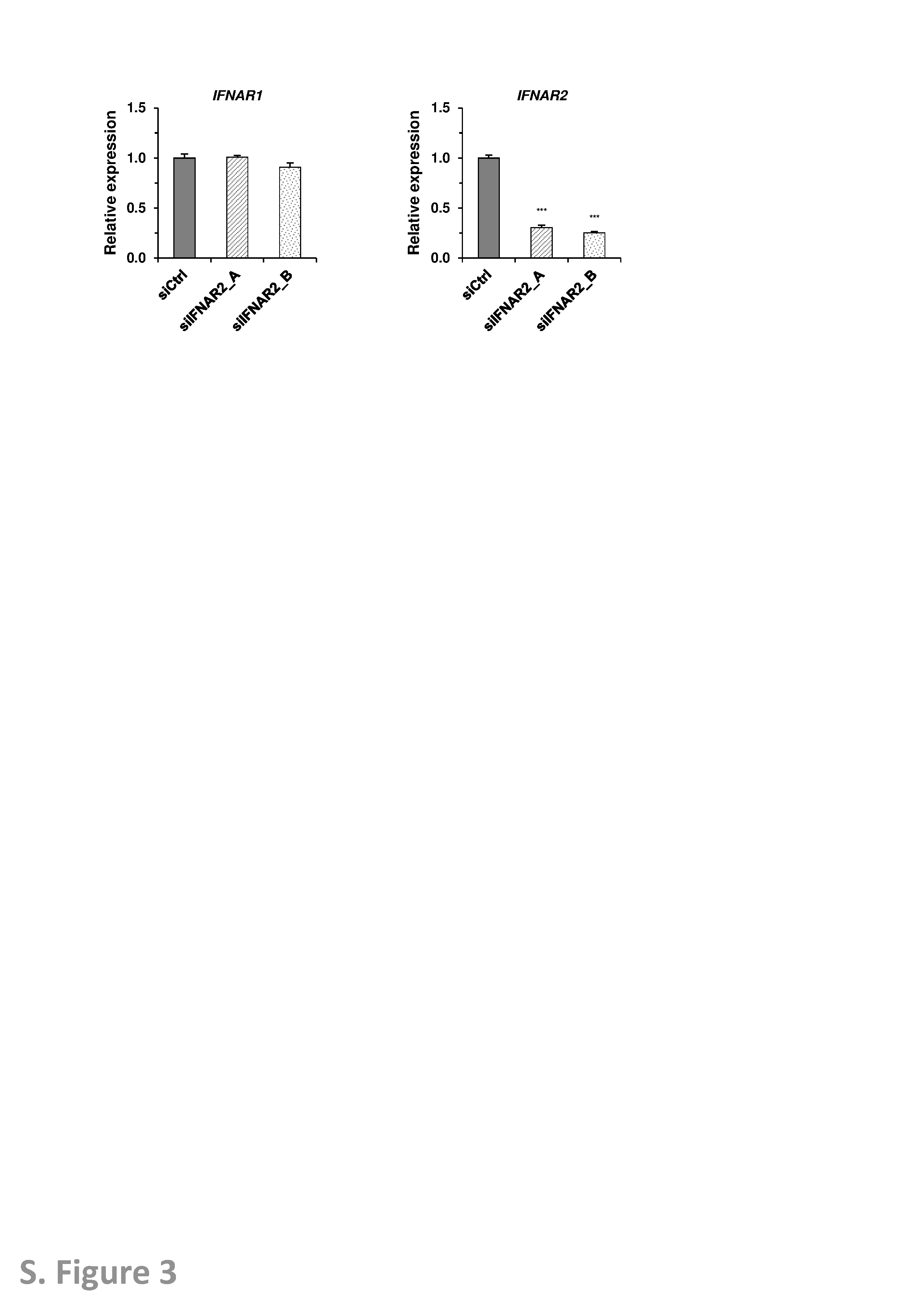

Supplement: Supplementary file 8 — S. Figure 3 [file 41420_2021_515_MOESM8_ESM.tif]
