# Supplementary material for: Epigenetic reprogramming promotes the antiviral action of IFNα in HBV-infected cells
Source: Cell Death Discov. 2021 Jun 2;7:130. doi: 10.1038/s41420-021-00515-y (PMC8170866; doi:10.1038/s41420-021-00515-y)
Supplement: Supplementary file 9 — SUPPLEMENTARY FIGURE LEGENDS [file 41420_2021_515_MOESM9_ESM.docx]

**SUPPLEMENTARY FIGURE LEGENDS**

**S. Figure 1. Assessment of IFNα and CDM-3008 cytotoxicity**

(**A**) Evaluation of IFNα and (**B**) CDM-3008 cytotoxic effects in HepG2-NTCP-C4 cells and REP-HepG2-NTCP cells. After IFNα and CDM-3008 treatment at the indicated concentrations for 9 days, cell viability was determined using an XTT assay. The data represent the means ± SD. Statistically significant differences in cell viability relative to that of the untreated cells were evaluated by t-test: *p < 0.05.

**S. Figure 2. *ISG20* and *APOBEC3G* methylation profiles**

(**A**) Comparison of *ISG20* and (**B**) *APOBEC3G* methylation levels between the REP-HepG2-NTCP cells and HepG2-NTCP-C4 cells. The COBRA data showed no methylation in the CpG sites upstream of the *ISG20* and *APOBEC3G* TSSs in either the control or reprogrammed HepG2-NTCP-C4 cells. The black arrows indicate the TSSs, and the black circles represent the methylation percentage for each analyzed CpG site (equivalent to 0% for these genes).

**S. Figure 3. Experimental silencing of *IFNAR2* in REP-HepG2-NTCP cells**

Relative expression levels of *IFNAR1* and *IFNAR2* were measured by real-time quantitative PCR 48 hours after transfection. Two distinct siRNAs were used to target *IFNAR2* (siIFNAR2_A and siIFNAR2_B). A scrambled siRNA was used as a negative control (siCtrl). *IFNAR2* knockdown did not affect *IFNAR1* expression. The histograms represent the means ± SD. Statistically significant differences in gene expression related to siCtrl-transfected cells were evaluated using a t-test: ***p < 0.001.
